# Bovine Viral Diarrhoea Virus Infection Disrupts Uterine Interferon Stimulated Gene Regulatory Pathways During Pregnancy Recognition in Cows

**DOI:** 10.3390/v12010001

**Published:** 2019-12-18

**Authors:** Zhangrui Cheng, Laura E Brown, D Claire Wathes

**Affiliations:** Department of Pathobiology and Population Sciences, Royal Veterinary College, Hawkshead Lane, Hatfield AL9 7TA, UK; lb00266@surrey.ac.uk (L.E.B.); dcwathes@rvc.ac.uk (D.C.W.)

**Keywords:** BVDV, endometrium, pregnancy recognition, interferon stimulated gene regulators, cows

## Abstract

In cattle, conceptus-derived interferon tau (IFNT) is the pregnancy recognition (PR) signal. Our previous studies showed that non-cytopathic bovine viral diarrhoea virus (ncpBVDV) infection inhibited IFNT-induced interferon stimulated gene (ISG) expression, potentially causing early embryonic death. This study investigated the effect of bovine viral diarrhoea virus (BVDV) infection on upstream regulatory pathways of ISG production using an established PR model. Uterine endometrial cells from 10 apparently healthy and BVDV free cows were cultured and treated with 0 or 100 ng/mL IFNT for 24 h in the presence or absence of ncpBVDV infection. Microarray and pathway analysis were used to determine the IFNT-induced upstream regulators. Expression of the genes associated with the identified pathways were quantified with qPCR. IFNT challenge activated the signalling pathways associated with IFN receptors, JAK1/TYK2, IRFs and STATs and ncpBVDV infection inhibited the activation of IFNT on this pathway. Inhibition of this upstream signalling pathway may thus reduce ISG production to disrupt maternal PR. In addition, the reduction of uterine immunity by ncpBVDV infection may predispose the animals to uterine infection, which in turn impairs their reproductive performance. This provides a mechanism of how BVDV infection leads to early pregnancy failure in cows.

## 1. Introduction

In cattle populations, embryonic mortality rates can reach up to 40% [1,2]. Between 2000 and 2006, a decline in reproductive performance was estimated to cause losses of up to £100 per cow per year in the United Kingdom [3,4]. The causes are complicated and not entirely understood [5,6]. Pregnancy recognition (PR) failure is one important cause for embryonic death and poor reproductive performance. The majority of embryonic mortality (70–80%) occurs before day 16, when maternal PR takes place [1,7,8,9]. Failure of PR in a timely fashion will be followed by regression of the corpus luteum, which will in turn causes loss of the embryo.

Interferon tau (IFNT) is released from the trophectoderm of the bovine conceptus between about days 8–25 of gestation. When it reaches a sufficient threshold by day 16, maternal PR (MPR) is initiated, including maintenance of the corpus luteum and development of a receptive environment in the uterus [10]. IFNT acts in a paracrine manner on the uterine endometrium in concert with the continued production of luteal progesterone to cause various changes in type I interferons, cytokines, prostaglandins (PGs) and growth factors and their receptors, which play important roles in remodelling the endometrium and developing a receptive environment for conceptus implantation [8,10,11,12,13]. Among these factors, type I interferon stimulated genes (ISGs) play crucial roles [11]. During IFNT-initiated MRP, numerous uterine genes (over 500) were differentially expressed with the greatest upregulation attributed to a group of pregnancy-specific ISGs [8,10,12,13]. The mechanisms determining how these ISGs act on MPR in vivo and implantation are not fully understood, but it has been suggested that upregulation of ISG production plays a crucial role in the modulation of uterine immunity, stromal remodelling, stimulating hyperplasia of the endometrial glands and development of the uterine vasculature [11,14].

There is strong evidence to suggest that bovine viral diarrhoea virus (BVDV) infection can disrupt pregnancy. BVDV is a positive sense single stranded (SS) RNA virus belonging to the genus *Pestivirus* and family *Flaviviridae*. Despite the introduction of control and eradication programmes in some countries, it remains endemic worldwide with major economic impact [15,16,17,18]. A systematic review using data derived from 15 countries for a period of 30 years showed that the direct losses were up to 687 USD per animal with higher losses of around 25 USD in naïve dairy than in beef cows [19]. Reproductive losses are one of the consequences of this disease. BVDV infection affects all stages of pregnancy, especially the early period [15,20,21].

BVDV exhibits vertical transmission from mother to foetus, potentially resulting in the birth of a persistently infected (PI) calf, which can subsequently spread the disease amongst the herd. Infection between animals is generally via the oronasal route, but direct transmission to the reproductive tract via semen or embryo transfer is also possible. Once acquired, the virus has a broad tissue tropism, and can replicate in many tissues, including the female reproductive system [20,22]. In support of this, our recent studies using an in vitro model of PR illustrated that BVDV inhibited bovine uterine defence systems to facilitate its own survival [23,24]. As expected, IFNT treatment alone significantly stimulated ISG expression in endometrial cell cultures, but concurrent BVDV infection significantly inhibited this stimulatory effect for 15 of the 17 ISGs tested (*ISG15*, *HERC5*, *USP18*, *DDX58*, *IFIH1*, *IFIT1*, *IFIT3*, *BST2*, *MX1*, *MX2*, *RSAD2*, *OAS1Y*, *SAMD9*, *GBP4* and *PLAC8*) [24]. This suggests a potential mechanism by which BVDV infection impairs PR and reproductive performance.

ISG transcription is regulated by IFNT induced pathways involving a signal transducer and activator of transcription (STAT) 1 and STAT2. IFNT binds to a common type I IFN receptor composed of interferon alpha and beta receptor subunit 1 (IFNAR1) and subunit receptor 2 (IFNAR2) subunits. This initiates cell signalling via the Janus activated kinases (JAKs) and tyrosine kinase 2 (TYK2) pathways [25,26]. This pathway allows phosphorylation of STAT1 and STAT2 which dimerise and the subsequent formation of interferon stimulatory gene factor 3 gamma (ISGF3G) which includes the STAT1:STAT2 heterodimer and interferon regulatory factor 9 (IRF9). ISGF3G can then travel to the nucleus and activate interferon stimulated response elements (ISREs), inducing the transcription of ISGs [27].

In the present study, we wished to determine how ncpBVDV infection could prevent the upregulation of ISG within uterine endometrial cells in response to the PR signal IFNT. We, therefore, focussed on intracellular signals known to be initiated following activation of the type 1 IFN receptor in the endometrium. This involved the pathway of JAK/TYK2-IRF-STAT1 and STAT2 which acted as upstream regulators of ISG synthesis.

## 2. Materials and Methods 

Reagents and consumables were purchased from Sigma-Aldrich (Poole, Dorset, UK) or Thermo Fisher Scientific (Paisley, UK) unless otherwise stated. All culture media contained 50,000 units/L penicillin and 50 mg/L streptomycin and were certified BVDV free. BVDV free foetal bovine serum (FBS; PAA, Somerset, UK) was used for the cell isolation and culture. Cell isolation and culture procedures were carried out under sterile conditions. The cells were cultured at 37 °C with 5% CO_2_.

### 2.1. Animals, Cell Isolation and Culture

Fresh and apparently health uteri from cows in the early luteal phase of their oestrous cycle were collected at the local abattoir. Potential BVDV contamination was tested with a PCR method used in our laboratory with the primer pair: forward (5’-ATGCCCWTAGTAGGACTAGCA-3’; position 108–128) and reverse (5’-TCAACTCCATGTGCCATGTAC-3’; position 395-375) [23,28] and a BVDV-positive control prepared using the pT7Blue-2 blunt vector, linearized (Novagen, Cambridge, MA02139, USA). The expected sequence covered the highly conserved 5ʹ non-coding/non-structural coding regions of the pestivirus BVDV genome strain NADL [28]. The testing system also included a reverse-transcription-negative control and a reference gene *ACTB* (see Table 1 for its primers). The uteri from 10 BVDV-free cows were selected for the experiments.

Uterine endometrial cells (a mixture of primary epithelial and stromal cells) were isolated and cultured using the methods established in our laboratory [23,24]. Briefly, strips of intercaruncular endometrium were dissected and put into Dulbecco’s Modified Eagle’s Medium/Nutrient Mixture F-12 Ham (DMEM/F12 medium) (Sigma). They were cut into 1 mm^3^ cubes using a mechanical tissue chopper (McIIwain Laboratory Engineering, Guilford, Surrey, UK). About 40 g of the chopped tissue was mixed with 60 mL digestive solution split into two 50 mL sterile vials. The digestive solution contained 100 mg bovine serum albumin (BSA, Sigma), 50 mg trypsin III (Worthington, Lakewood, NJ 08701, USA) and 50 mg collagenase A (Roche, Welwyn Garden City, UK) per 100 mL of Hanks’ balanced salt solution (HBSS; Sigma). After incubation for 90 min at 37 °C with 5% CO_2_ with manual mixing every 30 min, the cell suspension was filtered through 100 µm mesh into 50 mL falcon vials containing 10% FBS and 3 µg/mL trypsin inhibitor (Sigma) with HBSS added to 50 mL and centrifuged at 100× *g* at 10 °C for 10 min. After two repeats of the aforementioned washing procedures, the cells were suspended with the culture medium (DMEM/F12 medium with 10% FBS) and plated in 24-well IWAKI micro plates (Scitech DIV, Asahi Techno Glass, Japan) at 2 mL per well containing 0.5 × 10^5^ cells (day 1). Culture medium was changed every 48 h to allow the cells to grow. Contamination of immune cells was determined by immunocytochemical staining validated in our laboratory [29].

### 2.2. Experimental Protocols

The ncpBVDV (Pe515nc strain) was isolated from a cow diagnosed with mucosal disease and virologically cloned as non-cytopathogenic virus by the BVDV Research Group (Royal Veterinary College, UK). The virus stock was propagated to achieve a 50% tissue culture infective dose (TCID_50_) of 5 × 10^5^ per ml following the method previously used in our group [30]. Cells from each cow were grown in two 24-well plates as described previously (day 1), one for ncpBVDV infection and another for a non-infected control, to prevent cross-contamination. The BVDV inoculation was carried out on day 4 of the cell culture when the cells reached about 70% confluence. To infect the cells with ncpBVDV, the wells were inoculated with medium containing Pe515nc BVDV at a multiplicity of infection (MOI) of 0.1 for 3 h in 0.25 mL of maintenance medium (MM, DMEM/F12 medium with FBS reduced to 5% to prevent overgrowth of the cells). For the cells designated as the non-infected controls, 0.25 mL MM was added to each well following the aforementioned procedures. The volume in all wells was made up to 1 mL with MM and the medium was changed after two days. IFNT treatment was carried out 4 days after infection (day 8). For the wells specified for IFNT treatment, the medium was replaced with 1 mL MM containing 100 ng IFNT (recombinant ovine IFNT, Cell Sciences, Canton, USA) and incubated for 24 h. Thus, the cells from each cow were taken as a batch and subjected to four treatments: Control (CONT), IFNT, ncpBVDV and ncpBVDV+IFNT. The cells from each treatment group in each cow (6 wells) were pooled for total RNA extraction using RNeasy Mini kits (Qiagen, Manchester, UK) following the supplier’s protocol and stored at –80 °C for PCR and qPCR assays. Another set of the treated cells were lysed with Buffer RLT (Qiagen) and stored at –80 °C for STAT2 protein assay.

### 2.3. Assessment of BVDV Cell Infection and Cell Viability

The ncpBVDV infection in bovine endometrial cells was assessed using the PCR method with the extracted RNA (see above) and an indirect enzyme immunostaining as described previously [30]. After the cells were exposed to all treatments, their viability was estimated with an MTS reduction assay following the supplied protocol [29].

### 2.4. Primer Design and PCR

In this study, conventional PCR was used to check specificity of the primers and to produce the gene amplicons for preparing standard curves used in the absolute qPCR. DNA sequences for all primers were obtained for GenBank (https://www.ncbi.nlm.nih.gov/gene/) and the primers were designed using a Primer3 version 4.1.0 (http://bioinfo.ut.ee/primer3/). The primer sequence information is shown in Table 1. The primers were made by Eurofins Genomics (Ebersberg, Germany).

The amplicon length (100–200 bp) and melting temperature (around 60 °C) were optimized according to the recommendation by PCR Biosystems (London, UK) who supplied the reagents for cDNA synthesis (RT) and qPCR. The alignment specificity and quality were confirmed with the Blast tool (https://www.ncbi.nlm.nih.gov/tools/primer-blast/).

The potential genomic DNA contamination in the RNA extract was eliminated with RQ1 RNase-Free DNase kit (Promega Corporation, Madison, WI, USA) based on the supplied protocol. 1 µg of the treated RNA was reverse transcribed into cDNA using a cDNA synthesis kit (PCRBiosystems, London, UK) according to the supplier’s protocol. To minimize potential variation, a mastermix of reagents were made and all samples and standards were accommodated in a single PCR plate under the same conditions. The resulting cDNA was diluted to 100 µl. The conventional PCR for the candidate genes was carried out using the method and reagents described previously [24]. One proportion of the resulting product was subjected to electrophoresis on a 2% (w/v) agarose gel to confirm the primer specificity and another proportion was purified using a QIAquick PCR purification kit (Qiagen) for preparation of the standard curve and annealing temperature optimization, used in the qPCR assay. The concentration and quality of the purified cDNA was determined with a NanoDrop ND 1000 spectrophotometer (NanoDrop, Technologies Inc, Wilminton, DE, USA).

### 2.5. Microarray Hybridization and Analysis

To identify the upstream regulation of the ISG signalling pathways, the extracted RNA from the cells treated with IFNT and CONT in four cows per group were selected for microarray hybridization using the Affymetrix Bovine Gene 1.1 ST platform (Affymetrix, Santa Clara, USA) following the methods described previously [29]. Microarray hybridization and scanning were performed in Edinburgh Genomics (The Roslin Institute, University of Edinburgh, Easter Bush, Midlothian, UK) using a one-round amplification (one-cycle target labeling) protocol with the GeneTitan instrument (Affymetrix) following their protocols (http://genomics.ed.ac.uk/resources/protocols). The data were processed and analyzed with GeneSpring GX software V12.5 (Agilent Technolgy, Santa Clara, USA) using the annotation files provided. RMA16 with quantile normalization and median polish was used for background correction, normalization and summarization. Samples were paired within the same cow and a paired t-test with *p*-values adjusted via Benjamini–Hochberg false discovery rate was used to compare the differences between the treatment groups. Statistical significance was considered at *p* < 0.05. The genes with an absolute fold change > 1.25 between the IFNT and CONT groups were selected for further analysis.

### 2.6. Ingenuity Pathway Analysis

The differentially expressed genes identified in the aforementioned microarray analysis were uploaded onto the server of Ingenuity Pathway Analysis (IPA; Qiagen) to map to the genomic database and to analyse the upstream regulation and networks. The criteria uploaded included gene symbols, fold changes and adjusted *p* values.

### 2.7. qPCR Analysis for Gene Expression

Based on the results generated in the aforementioned upstream regulation and network analyses, 10 target genes (*IFNAR1, IFNAR2, STAT1, STAT2, IFR7, IFR9, JAK1, TYK2, PIAS2* and *IFNG*) and 4 potential reference genes (*GAPDH, RPL19, ACTB* and *18SrRNA*) were selected for expression quantification using the methods described previously [24]. Before qPCR assay, the optimal annealing and amplicon-specific melting temperature were determined using the gradient function built in the qPCR system (CFX96 Real-Time qPCR, Bio-Rad Laboratories). There were eight identical reactions containing 0.25 ng of the DNA standard, 10 µL SyGreen Mix (PCRBiosystems), 0.8 µL of each, 10 µM forward and reverse primer and nuclease-free water added up to 20 µL in each reaction. We used an absolute qPCR approach for quantification of the gene expression, in which each assay included a standard curve with eight concentrations ranging from 1 to 1 × 10^−7^ ng/mL, no template control (NTC) and sample cDNA from RT. All these were prepared in duplicate and fitted into one PCR plate (StarLab, Milton Keynes, UK) with the same mastermix of reagents. Two step amplification was used based on the recommendation by the qPCR reagent supplier (PCRBiosystems). Each qPCR reaction contained 5 µL of standard, sample cDNA or nuclease-free water (NTC), 10µL SyGreen Mix (PCRBiosystems), 0.8 µL of 10 µM forward and reverse primer and 3.4 µL nuclease-free water. The qPCR assay was carried out in a Bio-Rad CFX Real-Time qPCR system (Bio-Rad, Hercules, USA), including a Tag activation at 95 °C for 2 min, 38 cycles of denaturing at 95 °C, and annealing/extension for 30 s at the optimized temperatures listed in Table 1. This was followed by melting curve analysis to check the quality of the qPCR assays. The results were analyzed using the CFX Manager Software package (Bio-Rad). The limit of quantification was 1 × 10^−6^ to 1 × 10^−7^ ng/mL for all tested genes.

### 2.8. STAT2 Protein Assay

The cell lysate from three wells of the same treatment was collected and pooled with a volume of 0.6 mL after the treatments. A proportion of lysate (0.1 mL) was further diluted 20 times and centrifuged at 4 °C and 10,000× *g* for 20 min. The supernatants were collected for determining STAT2 protein using an enzyme-linked immunoassay (ELISA) kit purchased from Insight Biotechnology (Middlesex, UK) following the supplier’s protocol. The total proteins in the cell lysates were determined with a NanoDrop ND 1000 spectrophotometer. The concentration was normalized to pg/mg protein.

### 2.9. Statistical Data Analysis

The mRNA expression values were expressed as fg/μg RNA. The four quantified house-keeping genes were loaded to GeNorm software (Ghent University Hospital Centre for Medical Genetics, Belgium). The gene with the smallest M values was selected as the stable gene. Analysis of variance (ANOVA) was carried out to test the differences in HK gene expression (as identified with GeNorm) between groups. The results showed that *GAPDH* was the most stable gene, therefore, the expression values of all target genes were normalized to the expression values of *GAPDH* as the target gene/*GAPDH*, described previously [24]. The experiment was based on a randomized block design, in which the cells isolated and cultured from each cow were subjected to all treatments (CONT, BVDV, IFNT and BVDV+IFNT) and 10 cows (batches) were used. Statistical data analysis was carried out for each gene separately using ANOVA via a linear mixed effect model built in SPSS 25 (Chicago, IL, USA), in which the four treatments were set as fixed effect and cow (batch) as random effect. The diagnostic plots for this model are provided in File S2 and the variance components in File S3. The level for statistical significance was set to *p* < 0.05. A significant difference was shown in ANOVA, Fisher’s Least Significant Difference (LSD) multiple comparisons were carried out to determine the differences between each treatment pair. Levene’s test was carried out to assess the homogeneity of variances across the groups. The results showed that the variances of IFNAR2 and IFNG were not homogeneous (File S4). The homogeneity could not be achieved with logarithmic and Box-Cox transformation. The data for these two genes were, therefore, analyzed using Welch’s ANOVA with Games-Howell multiple comparison.

## 3. Results

### 3.1. Validation of Cell Culture and BVDV Infection

Examination with PCR showed that all uteri selected for the present study were free of BVDV infection/contamination. For the groups ncpBVDV and ncpBVDV+IFNT, the cultured cells were successfully infected with the BVDV virus as confirmed with PCR and immunocytochemistry that was described previously [30]. The MTS reduction assay illustrated that no treatments altered the cell viability. We measured the expression of vimentin (*VIM*, a marker for stromal cells) and keratin 19 (*KRT19*, a marker for epithelial cells) in the treated cells and the results showed that both *VIM* (55704 ± 9052 fg/µg RNA) and *KRT19* (16945 ± 2262 fg/µg RNA) were well expressed in the cultured endometrial cells. There were no significant differences in expression of these genes or their ratios between all treatment groups (CONT, ncpBVDV, IFNT and ncpBVDV+IFNT) (*p* > 0.05) as tested with ANOVA. This indicated that there were reasonable populations of both endometrial epithelial and stromal cells in our cell culture systems and that neither ncpBVDV nor IFNT affected the cellular growth and population. Contamination of immune cells in the cultured cells was negligible (< 0.001%).

### 3.2. Identification of Upstream Regulatory Pathways

**Figure 1:**
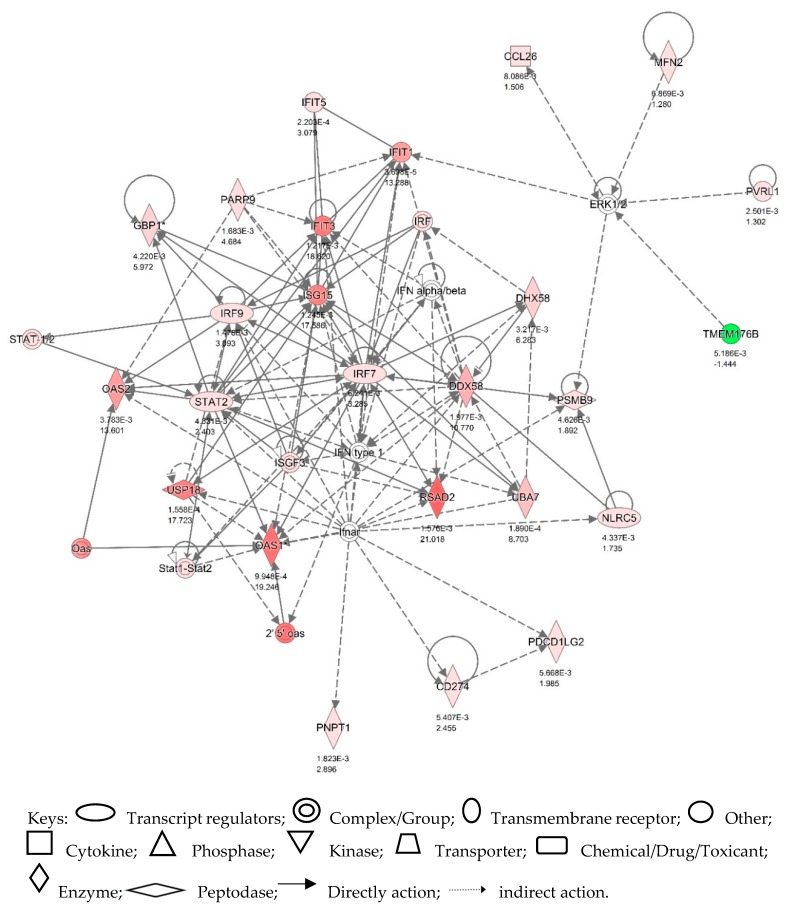
Network 1 showing STAT1 and STAT2—IRF signalling pathways in bovine endometrium which play central roles in regulating IFNT-initiated ISG production. The endometrial cells were isolated from freshly collected uteri of apparently healthy cyclic cows (*n* = 4) and cultured to confluence. The cells were challenged with 0 or 100 ng/mL IFNT for 24 h on day 8 of the cell culture. RNA was extracted from the treated cells and subjected to microarray hybridization using Affymetrix Bovine Gene 1.1 ST platform. The DEGs were uploaded onto the IPA for network analysis. The network is displayed graphically as nodes and edges (the biological relationship between expression of genes: red—upregulated, green—downregulated in IFNT vs. CONT in endometrial cells). The fold value (IFNT: CONT) and *p* values are indicated under each node. The shapes of nodes indicate the functional class of the gene product as shown in the key.

**Figure 2:**
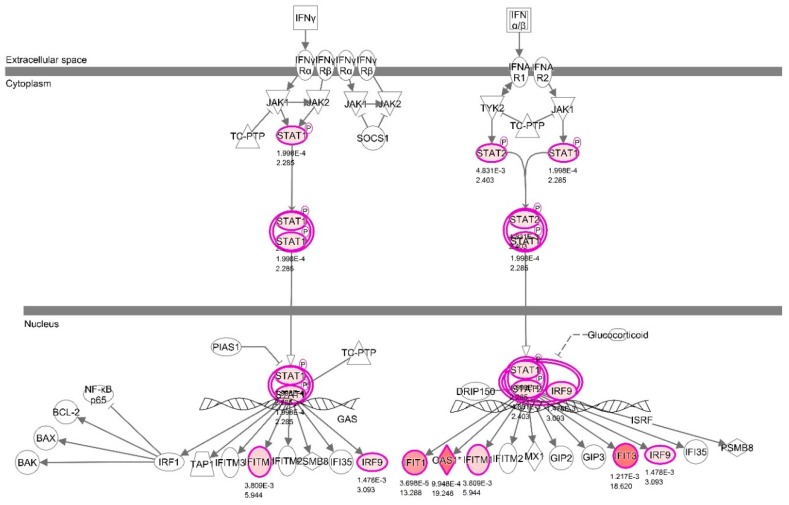
Canonical pathways showing the position of DEGs within interferon signalling pathways following IFNT challenge in bovine uterine endometrium. Refer to the legend of Figure 1 for details of the experimental design. The pathways show the biological relationship between the expression of genes: red—upregulated in IFNT vs. CONT in endometrial cells. The fold value (IFNT: CONT) and *p* values are indicated under each node. The purple lines represent the focus genes. The shapes of nodes indicate the functional class of the gene product as shown in the key in Figure 1.

With the criteria set up above, the analysis showed that there were 214 differentially expressed genes (DEG) between the IFNT and CONT groups (*p* < 0.05) (See File S1 for the complete list). These DEG were uploaded onto the IPA server for (i) network and (ii) upstream regulation analysis. The system built over 10 networks. Figure 1 shows Network 1 which centered on the interferon regulatory factors (IRF7, IRF9 and ISGF3), STAT1 and STAT2. This was the most significant network identified with 25 focus molecules and a score of 45. Figure 2 illustrates two canonical pathways constructed by IPA, which illustrates the position of those DEG which were known to feature in the IFN type 1 and type 2 signalling pathways.

The IPA upstream regulator analysis is based on prior knowledge of expected effects between transcriptional regulators and their target genes. It examined how many known targets of each transcription regulator were present in our dataset, and also compared their direction of change. This analysis identified over 200 potential upstream regulators, in which the top upstream regulators were associated with the ISG regulatory pathway of IRF-STAT1 and -STAT2 (Table 2). Their activation led to the production of many ISGs.

### 3.3. Determination of Reference Genes for Normalization

The effect of ncpBVDV, IFNT and their combination on the selected reference genes was determined with the absolute qPCR method and the results showed that all four selected reference genes were highly expressed in the cultured bovine endometrial cells. ANOVA showed that expression of *ACTB*, *RPL19* and *18SrRNA* was significantly affected by either IFNT or ncpBVDV treatment. *GAPDH* was, however, stably expressed between the treatment groups and GeNorm analysis showed that it had the smallest M values among the four tested reference genes. Therefore, the expression values of all ten candidate genes were normalized to *GAPDH* for the subsequent comparison of the treatment effects.

### 3.4. Effect of IFNT Alone on Candidate Gene Expression

**Figure 3:**
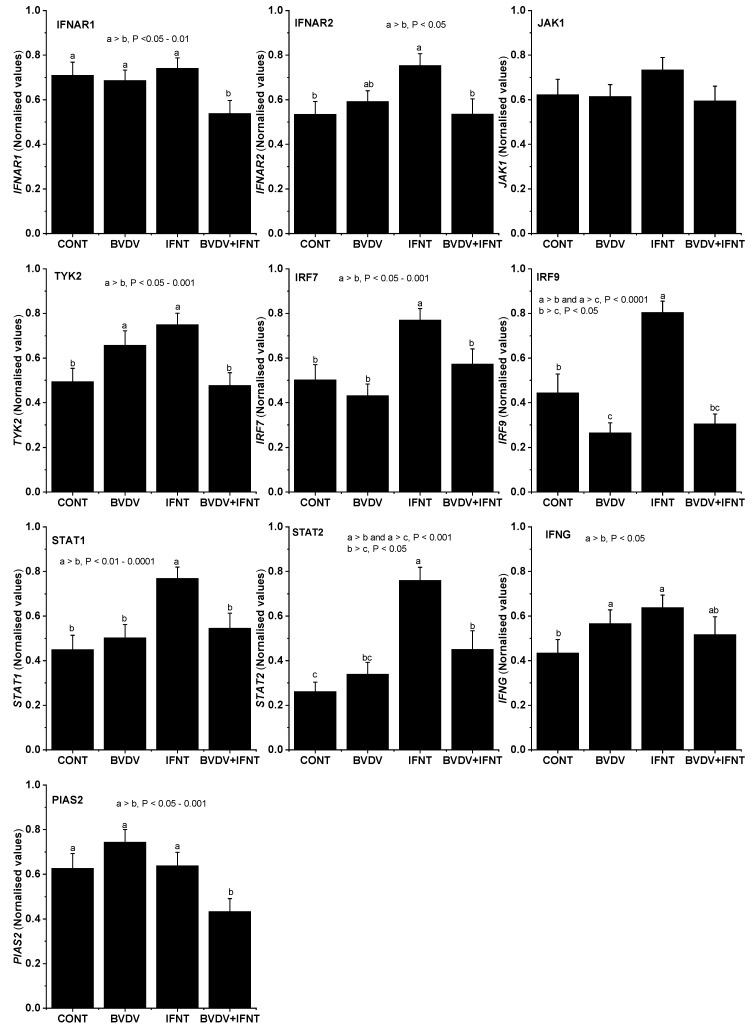
Effect of ncpBVDV, IFNT and their combination on expression of the genes associated with JAK/TYK2-STAT1 and STAT2–IRF signalling pathways in uterine endometrial cells in cows, measured with qPCR and normalised to *GAPDH* as described in the Methods. The columns labeled with different letters were significantly different and the *p* values are indicated in the graph for each gene.

**Figure 4:**
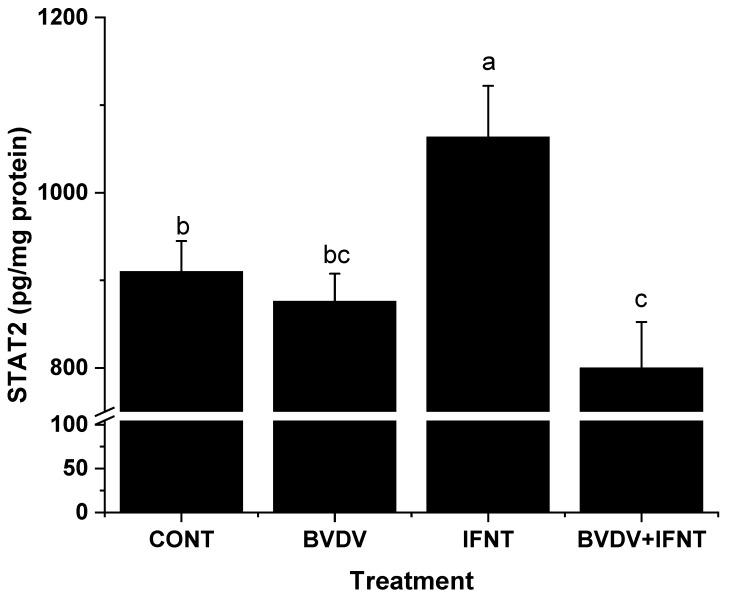
Effect of ncpBVDV, IFNT and their combination on the production of STAT2 protein measured with ELISA in lysates of the cultured bovine uterine endometrial cells treated with CONT and IFNT in the absence and presence of ncpBVDV infection. The concentration was normalized to pg/mg protein. a > b and a > c, *p* < 0.01; b > c, *p* < 0.05.

Treatment with 100 ng/mL IFNT for 24 h significantly increased the expression of *IFNAR2, TYK2, STAT1, STAT2, IRF7, IRF9* and *IFNG* (*p* < 0.05-*p* < 0.001), however, did not alter the expression of *IFNAR1* or *PIAS2. JAK1* expression was numerically higher than in the CONT cells but this failed to attain statistical significance (*p* > 0.05). The concentration of STAT2 protein was also significantly increased (*p* < 0.01) (Figure 3 and Figure 4).

### 3.5. Effect of BVDV Alone on Candidate Gene Expression

Treatment with Pe515nc BVDV for 5 days significantly increased the expression of *TYK2* and *IFNG* (*p* < 0.05), but did not alter the mRNA expression of *IFNAR1, IFNAR2, JAK1, STAT1, STAT2, IRF7*, *PIAS2* or the concentration of STAT2 protein. Expression of *IRF9* was reduced (*p* < 0.05) (Figure 3 and Figure 4).

### 3.6. Effect of BVDV on IFNT-Treated Cells

For all seven genes whose expression was increased by IFNT alone, the presence of BVDV infection prevented this rise (*IFNAR2, TYK2, STAT1, STAT2* (both gene and protein), *IRF7, IRF9* and *IFNG*) (*p* < 0.05–0.01). The combined treatment also reduced the expression of *IFNAR1* and *PIAS2* and the concentration of STAT2 protein in comparison with the control cultures (all *p* < 0.05). *JAK1* expression was not altered (Figure 3 and Figure 4).

## 4. Discussion

**Figure 5:**
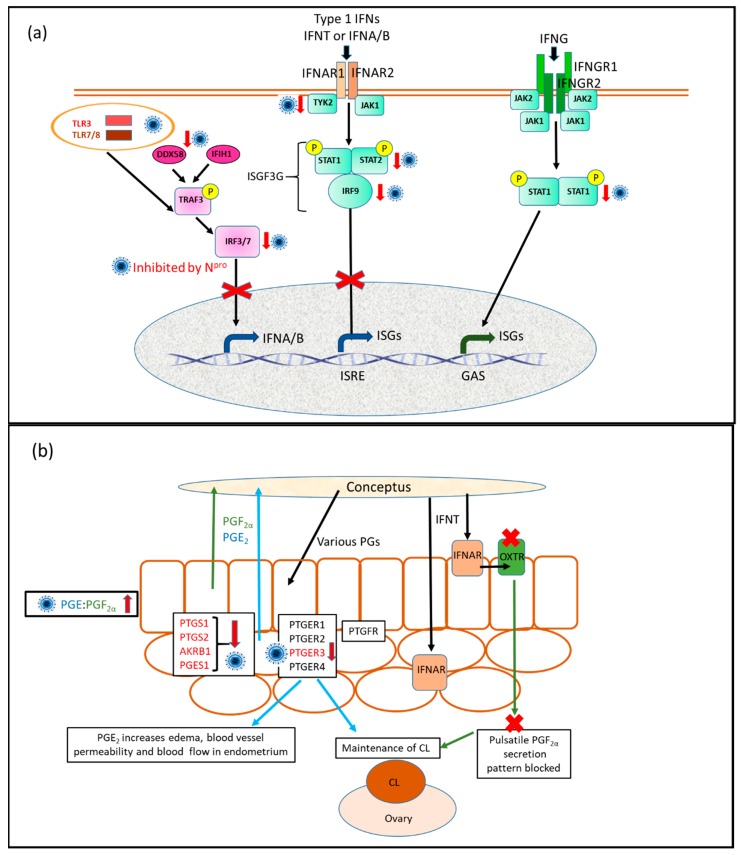
During pregnancy recognition (PR) in cattle conceptus produced IFNT increased expression of type I IFN-stimulated genes (ISGs) in the uterine endometrium and increased prostaglandin (PG) production facilitates these effects [13]. Our previous work showed that when endometrial cells were treated with both ncpBVDV and IFNT, then the increase in endometrial expression of most ISGs tested (which normally accompanies maternal PR) was strongly inhibited [24]. (**a**) The present study has extended these observations to investigate the upstream signalling pathways involved, notably the combined treatment inhibited expression of *TYK2*, *STAT1*, *STAT2* and *IRF9*. These are all crucial to signalling of type 1 IFNs through the IFNARs and would explain why upregulation of many ISGs inhibited ncpBVDV. Of these, only STAT1 is also required for signalling via IFNG and the IFNG receptor, therefore this pathway is likely to be less affected by ncpBVDV. Following uptake of BVDV into endometrial cells by endocytosis, the virus is initially incorporated into the endosome, where it may be sensed by TLR3 or TLR7/8. For replication to occur, viral RNA then needs to be released into the cytoplasm where it can also be detected by the cytoplasmic pattern recognition receptors DDX58 and IFIH1. These receptors signal via TRAF3 and IRF3 or IRF7 to increase cellular production of type 1 IFN [50]. We showed previously [24] that ncpBVDV could inhibit the expression of *DDX58* and *IFIH1*, while the present results found inhibition of IRF7. The BVDV protein N^pro^ targets IRF3 toward proteasomal degradation [44]. Therefore, this pathway is also inhibited by the presence of ncpBVDV, therefore preventing the host from responding to the virus by producing type 1 IFN. Note that the pictures of the virus indicate points in the pathway, which were downregulated in ncpBVDV infected cells. These effects may have been indirect. Overall, these combined changes induced in the endometrium by ncpBVD are likely to interfere with the normal pathways required for pregnancy recognition. (**b**) With respect to PG production, epithelial cells predominantly synthesise PGF_2α_ whereas stromal cells produce most of the PGE_2_ and the conceptus itself synthesises a variety of PGs including PGF_2α_, PGE_2_ and 6-keto-PGF_1α_. Both endometrial cells and the conceptus contain receptors for both PGE and PGF. One of the key actions of IFNT in maternal pregnancy recognition is to block the development of oxytocin receptors in the epithelium, therefore preventing PGF_2α_ from being released in large pulses, which can cause luteal regression [51]. Nevertheless, total PG production increases substantially in early pregnancy [52]. Our previous work [23] has shown that infection of endometrial cells with ncpBVDV alone increases PGE_2_ endometrial synthesis while decreasing PGF_2α_, thus increasing the PGE_2_:PGF_2α_ ratio. When endometrial cells were treated with both ncpBVDV and IFNT, expression of the main PGF synthase *AKRB1* was decreased, reducing synthesis of PGF_2α_ and endometrial expression of *PTGER_3,_* which was inhibited. These changes are likely to reduce the required PG signalling within the endometrium during PR and have been shown to act in synergy with IFNT [12,13].

Upregulation of uterine ISG production by IFNT released from the conceptus trophectoderm is a vital component of maternal PR in ruminants [11]. This effect has been observed both in vivo [8] and in vitro [24]. BVDV infection disrupts various stages of pregnancy, including early pregnancy (PR and implantation) [20]. Our previous studies showed that ncpBVDV infection inhibited or neutralised the stimulatory effect of IFNT on ISG production [24]. The ISG signalling pathway is controlled by its upstream regulatory pathway: JAK/TYK2-STAT1 and STAT2–IRFs. Our present results demonstrated that, in the uterine endometrial cells, IFNT treatment upregulated the genes at various levels of the JAK/TYK2-STAT1 and STAT2 –IRF signalling pathways, including IFN receptors, JAK1/TYK2, IRFs and STATs. Microarray and pathway analyses showed that these pathways played a central role in IFNT-initiated MPR in cows. Infection with ncpBVDV inhibited the activation of IFNT on this pathway. This provides part of the mechanism by which BVDV infection leads to PR failure in cows and is summarised in Figure 5. It should be noted that our results were obtained after 24 h stimulation with IFNT, thus, only represents one time point. During pregnancy recognition IFNT production by the conceptus begins around day 8 of gestation and terminates around day 25, once the corpus luteum has been maintained and implantation has begun [31]. The endometrial cells are therefore exposed continuously to IFNT for a 17-day period, which is likely to represent a differing situation to the induction of other type 1 interferons during an infection.

The JAK/TYK2-STAT-IRF signalling pathway is involved in many processes including immunity, cell division, cell death and tumor formation [32,33]. Its activation also leads to up-regulation of many ISGs required for MPR to develop a receptive environment in the uterus [11]. This pathway does not usually function autonomously but is regulated by various intrinsic and environmental stimuli. Activation starts with IFNT binding to the type I IFN receptor complex composed of IFNAR1 and IFNAR2. We found here that IFNT challenge stimulated *IFNAR2* but not *IFNAR1* mRNA expression, although in a previous study Forde et al. [8] did not observe differential expression of either the INF receptor subunit in a microarray comparison of uterine endometrium between cyclic and pregnant cows on Day 16 (See their File S2). Treatment with ncpBVDV alone did not affect mRNA expression of either receptor subunit but the stimulatory effect of IFNT was inhibited in the presence of ncpBVDV infection. As STAT—activating cytokine receptors lack intrinsic tyrosine kinase activity, they require receptor-associated cytoplasmic proteins from JAKs and TYK2 to fulfil their functions, including IFNT-induced phosphorylation, formation of intracellular tyrosine residues on the receptor and formation of STAT docking sites [32]. In the present study, both ncpBVDV infection and IFNT alone significantly stimulated *TYK2* mRNA expression but this did not happen when both treatments were combined, indicating potential disruption of MPR at this level of the pathway. Neither ncpBVDV infection nor IFNT alone altered *JAK1* mRNA expression. It was reported that *JAK1* mRNA expression on day 16 was slightly lower in pregnant than in cyclic cows (see Forde et al. File S2) [8]. Together this indicates that TYK2 rather than JAK1 is the possible target for both ncpBVDV and IFNT at this level of the pathway in bovine endometrium. It should be noted, however, that in the present study the samples were collected five days after the BVDV infection was introduced and 24 h after IFNT challenge. The experimental protocol was, therefore, not designed to detect the immediate direct effects of either treatment on these signalling pathways, such as phosphorylation of JAK1 and TYK2. Within this timeframe secondary effects were likely to have occurred, which may have led indirectly to the changes in gene expression which were detected.

IRFs regulate the transcription of IFNs and ISG expression and the signalling pathway of Toll-like receptors and collaborate with other co-acting transcription factors such as nuclear factor-kappaB (NF-kappaB). They bind DNA and exert their activating potential as homo- or heterodimers. They can form complexes with STATs to activate the key steps of the JAK-STAT-IRF signalling pathway [32]. During the MPR on day 16 of pregnant cows, *IRF7* and *IRF9* mRNA expression in uterine endometrium was upregulated by about six-fold compared with the cyclic cows [8], suggesting their important roles in maternal PR. Inhibition of IRF-dependent transcriptional regulatory mechanisms is associated with many immune diseases [34]. In the present study, we tested the effect of ncpBVDV infection and IFNT challenge on the expression of *IRF7* and *IRF9* mRNA. Consistent with the aforementioned in vivo study, *IRF7* and *IRF9* mRNA expression were both significantly induced by IFNT treatment. However, in the presence of ncpBVDV infection, the stimulatory effect of IFNT disappeared. Disruption of this upstream regulatory pathway of ISG signalling may affect MPR. In addition, in the cells infected with ncpBVDV alone, expression of *IRF7* was slightly reduced and that of *IRF9* was significantly inhibited. Reduced expression of the IRFs may lead to decreased innate immunity, which could predispose cows to uterine infections which in turn might impair their reproductive performance [34,35]. 

STAT1 and STAT2 play central roles in the JAK/TYK2-STAT-IRF signalling pathway. After phosphorylation on both IFNAR receptors and the STAT proteins by JAK1 and TYK2 in the cytoplasm, STAT1 and STAT2 together with IRF9 formed a regulatory complex–IFN-stimulated gene factor 3 (ISGF3). ISGF3 enters the nucleus and binds to DNA to cause transcription of many ISGs [32] (Figure 5a). We investigated the expression of *STAT1*, *STAT2* and *IRF9* in the File S2 of the previous report [8] and found that all of them were significantly upregulated in bovine endometrium during maternal PR. Our present data agreed with the aforementioned in vivo results, as both *STAT1* and *STAT2* genes and STAT2 protein were significantly upregulated by IFNT challenge in bovine uterine endometrial cells. However, ncpBVDV infection suppressed the stimulatory effect of IFNT. Phosphorylation of the STAT proteins is a necessary component of this pathway. This was not investigated in the present study, however, it would be interesting to determine in future whether this step was influenced directly by ncpBVDV. It was also reported that STAT1 exerts its effect on promoting ISG production via an ubiquitin ligase complex PARP9-DTX3L, which targets host histone H2BJ and viral 3C protease to control viral infection [36]. In the present study, IFNT challenge increased the expression of both *PARP9* and *DTX3L* by up to six-fold (*p* < 0.01) in bovine uterine endometrial cells compared with the cells without IFNT challenge (See File S1) and PARP9 was identified as an upstream regulator for ISG production. This suggested that the PARP9-DTX3L pathway may also play a role in MPR.

Protein inhibitors of activated STATs (PIAS) are a group of ubiquitin-like modifiers, which add a marker to many genes, including JAKs and STATs, leading to their inhibition [37]. They act to control gene expression and are considered as transcriptional co-regulators [38]. PIAS1 and PIAS2 play key roles in the pathogenesis of autoimmune and inflammatory diseases [39]. In our present study, ncpBVDV alone moderately increased the expression of *PIAS2* mRNA. This was expected as ncpBVDV may upregulate production of the inhibitors of the JAK/TYK2-STAT-IRF signalling pathway to block its activation. It was not, however, expected that the combination of ncpBVDV with IFNT would reduce *PIAS2* expression significantly compared with the other three groups as this might, in theory, increase production of ISGs whereas our previous study showed that this combination inhibited the production of most ISGs we tested [24]. The decrease in PIAS2 production was possibly not effective enough to offset the inhibitory effect of ncpBVDV on IFNT-induced gene expression or some other yet unknown mechanisms were involved.

In our present study, the IFNT-induced JAK/TYK2-STAT1 and STAT2-IRF signalling pathways were significantly inhibited in the presence of ncpBVDV infection. Its downregulation not only inhibited production of the ISGs required for both PR and antiviral signalling, however, would also be expected to disrupt other immune mechanisms within the uterus. Our previous study demonstrated that IFNT challenge stimulated the ISG-production to develop a pro-immune and antiviral environment in the uterus whereas ncpBVDV inhibited this stimulatory effect [24]. There are at least five main effector pathways of IFN-mediated antiviral responses as identified by a gene target study and ISGs have been shown to play crucial roles in four of them [36,40]. Activation of these pathways can block viral transcription and translation, degrade viral RNA and modify protein function to control all steps of viral replication [40]. In addition, the inhibition of IRF-dependent pathways disrupts NF-κB signalling and many other immune mechanisms and is associated with reduced immunity and increased susceptibility to bacterial infections [34]. Thus, by inhibiting those immune mechanisms, ncpBVDV can evade host detection and destruction to facilitate its own proliferation, maintenance and spreading, and increase the likelihood of a secondary bacterial infection, such as *Escherichia coli* or *Trueperella pyogenes,* thus becoming established in the reproductive system [29,30,41].

Many previous studies have investigated how infection with pestiviruses, including BVDV, can cause immunosuppression [42]. BVDV has a viral RNase E^rns^ glycoprotein, which inhibits IFN expression induced by extracellular viral RNA and an N^pro^ site to promote the degradation of the transcription factor IRF3 [42,43,44,45]. This prevents synthesis of type 1 IFNs as illustrated in Figure 5a. Our data are in accord with this, as the comparison of gene expression profiles between control endometrial cells and those treated with ncpBVDV alone failed to find evidence for increased expression of any type 1 interferon genes present on the array (*IFNAA*, *IFNA16*, *IFNAH*, *IFNB1*, *IFNB3*) in response to the virus [30]. In contrast to our results, however, previous studies have concluded that although there is clear evidence showing that infection with ncpBVDV can block the induction of type 1 IFN synthesis, the action of IFN is not compromised [42]. For example, Schweizer et al. [46] showed using cultures of both primary bovine turbinate cells and monocyte-derived macrophages that treatment of cells with IFN α/β after infection with a strain of ncpBVDV reduced the level of viral RNA less than threefold compared to non-IFN-treated cells. In contrast, a very much greater reduction in virus occurred if the IFN was added prior to infection. Another series of studies aimed at determining how BVDV may induce foetal immune tolerance and the birth of PI calves has, however, concluded that the action of any type 1 IFN present is not affected [47,48,49]. Upregulation of the type 1 IFN pathway genes including ISGs in response to BVDV were reported in both the maternal white blood cells and in a variety of foetal tissues. These results clearly differed from ours as reported here and previously [24] using endometrial cells, in which production of most ISG in response to IFNT was inhibited by infection with type 1 ncpBVDV (although not entirely absent). The models used were, however, very different. The in vitro studies used different cell types, while in the in vivo experiments mid to late pregnant heifers were inoculated with ncpBVDV type 2 and then responses were measured at various time intervals after infection. It should be noted that the foetus is immunologically immature at this stage and also that our cultures did not contain white blood cells. Therefore, both the tissues and the strains of BVDV tested differed in these various experiments. According to Peterhans and Schweizer [42] responses of IFN signalling system to BVDV infection are known to differ between organs and cell types. Our test model relates to maternal PR in which the conceptus is producing significant amounts of IFNT over at least a two-week period and it is the direct action on the maternal endometrial cells that matters. This suggests that the local immune mechanisms operating in the uterus during the establishment of pregnancy differ to those involving either the maternal white blood cells in circulation or the developing foetus later in gestation.

Our work has focussed mainly on type 1 interferons, however, type II interferon (IFNG) also plays an important role in adaptive and innate immunity against both bacterial and viral infections. One of its important signalling pathways is also through the action of JAK1 and STAT1 to form an IFNG-activated factor [32] (see Figure 5a). In our present study, we used IFNG as a marker for general immune responses. Neither *JAK1* nor *STAT1* expression was influenced by the treatment with ncpBVDV alone whereas *IFNG* expression was enhanced. The increased IFNG expression suggests that the cultured bovine endometrial cells retained some ability to offer a normal immune response to the viral infection.

With regard to the establishment of pregnancy, experiments performed primarily in sheep showed that prostaglandins (PGs) produced by both the endometrium and the conceptus itself were important in the establishment of pregnancy, and interacted with IFNT in regulating endometrial gene expression [12,31]. In particular, ewes treated with IFNT had increased activity of the key enzyme prostaglandin synthase, associated with higher levels of PGE_2_, PGF_2α_ and 6-keto-PGF_1α_ in the uterine lumen. Furthermore, induction of a number of IFNT stimulated genes were inhibited when the animals were co-treated with the PG synthase inhibitor meloxicam. This indicated that PGs act in concert with IFNT to promote development of the conceptus. We, therefore, previously investigated the effects of ncpBVDV and IFNT on PG synthesis in cultured bovine endometrium [23]. This showed that ncpBVDV alone increased PGE_2_ endometrial synthesis while decreasing PGF_2α_, so increasing the PGE_2_:PGF_2α_ ratio. This was achieved by increasing expression of both *PTGS_1_* and *mPGES_1_*, and would be predicted to cause inflammatory changes such as stromal oedema and to promote maintenance of the corpus luteum in the absence of a conceptus. When endometrial cells were treated with both ncpBVDV and IFNT, the main PGF synthase *AKRB1* was decreased, reducing synthesis of PGF_2α_ and endometrial expression of *PTGER*_3_ was inhibited. These changes are therefore likely to act in concert with the alterations in the IFNT signalling pathway as described here to inhibit PR in cattle infected with ncpBVDV (Figure 5b). 

## 5. Conclusions

In conclusion, our study showed that JAK/TYK2-STAT1 and STAT2–IRF signalling pathways play central roles in MPR initiated by IFNT in cows and that ncpBVDV infection inhibited the activation of IFNT on this pathway. Our study design did not distinguish between direct and indirect effects of the virus so it is possible that the reduction in expression or activity of IRF-3/IRF-7 by viral N^pro^ might inhibit the positive feedback loop for IFN type-I expression that might be initiated by the addition of IFNT. We demonstrated here that inhibition of this upstream signalling pathway for ISG production was likely to disrupt MPR. In addition, the reduction of uterine immunity by ncpBVDV infection may predispose the animals to uterine infection, which in turn would impair reproductive performance. This provides a mechanism for how BVDV infection leads to PR failure in cows.

## Figures and Tables

**Table 1:** Oligonucleotide primer sequence information.

Gene	Primer Sequence (5′-3′)	GenBank Accession	Product Length (bp)	Annealing (ᵒC)
***ACTB***	Forward: GAAATCGTCCGTGACATCAA Reverse: AGGAAGGAAGGCTGGAAGAG	NM_173979.3	182	62.8
***GAPDH***	Forward: GGTCACCAGGGCTGCTTTTA Reverse: TTCCCGTTCTCTGCCTTGAC	NM_001034034.2	147	61.4
***RPL19***	Forward: TCGATGCCGGAAAAACAC Reverse: ATTCTCATCCTCCTCATCCAG	NM 001040516	119	59
***18SrRNA***	Forward: CGGCGACGACCCATTCGAAC Reverse: GAATCGAACCCTGATTCCCCGTC	AY779625	99	64.5
***IFNAR1***	Forward: CATGTCAGTGTTGGTGCTTCAG Reverse: ACACAATACACAGTCAGCGGTT	NM_174552.2	173	60.2
***IFNAR2***	Forward: AAAAGTGGCTACCGTGGAAGTC Reverse: TGAAGTGGTGGAAGTTGGACAC	NM_174553.2	169	60.2
***IRF7***	Forward: AAGTGCAAAGTCTACTGGGAGG Reverse: CAAGTAGATGGTGTAGTGCGGG	XM_015461322.1	180	60.2
***JAK1***	Forward: TGAGAACGAGTGTCTTGGGATG Reverse: GGTGAGAAGGTTCCTCTGTCTG	XM_010803450.2	154	62.8
***PIAS2***	Forward: CCGAGAATTGTATAGACGCCGA Reverse: AGAGGACGGAGAATGAGGTGTA	XM_010825599.2	178	61.4
***TYK2***	Forward: GAAGTTCCCTATCGAGCTCCAG Reverse: GTGTGACGATGAGGTTGGAGAT	XM_005208739.3	185	62.8
***IRF9***	Forward: TACCATCAAAGCGACCCCAC Reverse: AAGTCTAAACGGCCAGCTCC	XM_010808904.2	151	60.2
***STAT1***	Forward: GTCTCAATGTGGACCAGATGA Reverse: TCATTCCAGAGAGCAAGCAGG	XM_005202573.3	187	60.2
***STAT2***	Forward: TCCTGCTGCGCTTTAGTGAA Reverse: TCCTCCGTGAGCATCTGGTA	XM_005206561.3	169	62.8
***IFNG***	Forward: ATGCAAGTAGCCCAGATGTAGC Reverse: CTCAGAGCTGCCATTCAAGAAC	NM_174086.1	215	62.8

**Table 2:** Selected top upstream regulators for the genes identified in File S1, which were differentially expressed between the control cells and those treated with IFNT *.

Upstream Regulator	Predicted Activation State	Activation z-Score	*p*-Value
IRF7	Activated	5.344	7.22 × 10^−33^
IFNA2	Activated	4.958	8.72 × 10^−26^
IFNL1	Activated	4.439	2.62 × 10^−25^
IFNAR	Activated	4.415	8.00 × 10^−24^
ACKR2	Inhibited	−3.742	9.89 × 10^−22^
IFNG	Activated	5.923	2.76 × 10^−19^
TRIM24	Inhibited	−4.186	5.34 × 10^−19^
Interferon alpha	Activated	4.914	2.10 × 10^−17^
IFN Beta	Activated	3.938	8.29 × 10^−17^
TLR3	Activated	2.959	1.29 × 10^−16^
IRF3	Activated	4.155	8.61 × 10^−16^
IRF1	Activated	4.185	9.64 × 10^−16^
MAPK1	Inhibited	−4.482	1.15 × 10^−15^
IRF5	Activated	3.528	4.26 × 10^−15^
EIF2AK2	Activated	3.687	4.68 × 10^−14^
DDX58	Activated	2.481	8.59 × 10^−14^
DNASE2	Inhibited	−2.200	4.94 × 10^−13^
NKX2-3	Inhibited	−4.359	5.42 × 10^−13^
STAT2	Activated	2.567	2.50 × 10^−12^
STAT1	Activated	3.970	2.91 × 10^−12^
IFNA1/IFNA13	Activated	3.258	3.40 × 10^−12^
IFN	Activated	3.397	2.84 × 10^−11^
IFNAR1	Activated	2.592	5.57 × 10^−11^
PAF1	Activated	3.000	1.78 × 10^−10^
IRF9	Activated	1.951	4.50 × 10^−10^
STAT3	Activated	1.506	7.44 × 10^−10^
PARP9	Activated	2.425	1.51 × 10^−9^
IFN type 1	Activated	2.047	1.71 × 10^−9^
IFNAR2	Activated	2.236	2.99 × 10^−9^
UBA7	Inhibited	−1.992	2.33 × 10^−8^
PTGER4	Inhibited	−2.137	1.11 × 10^−7^
TGM2	Activated	2.514	2.08 × 10^−7^
DOCK8	Activated	2.828	2.74 × 10^−7^
TICAM1	Activated	3.120	2.86 × 10^−7^
IRF2	Inhibited	−1.597	1.99 × 10^−6^
IL1B	Activated	3.057	3.16 × 10^−6^
ISGF3			6.41 × 10^−5^
JAK1	Activated	2.219	3.71 × 10^−5^
STAT6	Inhibited	−0.956	1.78 × 10^−4^

* The upstream regulators were selected according to the activation z-score, *p* values derived from Fisher’s exact test and the relevance. The endometrial cells were isolated from freshly collected uteri of apparently healthy cyclic cows (*n* = 4) and cultured to confluence. They were challenged with 0 or 100 ng/mL IFNT for 24 h on day 8 of the cell culture. RNA was extracted from the treated cells and subjected to microarray hybridization using Affymetrix Bovine Gene 1.1 ST platform. The DEGs were uploaded on to IPA for upstream regulator analysis. The Z-score is a statistical measure of the match between expected relationship direction and observed gene expression.

## Supplementary Materials

The following are available online at https://www.mdpi.com/1999-4915/12/1/1/s1, Files S1: Differential expression of the genes between the bovine uterine endometrial cells treated with interferon tau and control, Files S2: Diagnostic plots for ANOVA with linear mixed effect model, Files S3: SPSS output of Variance components via linear mixed effect model, Files S4: Test of homogeneity of variance.

## References

[1] Diskin M.G., Parr M.H., Morris D.G. (2011). Embryo death in cattle: An update. Reprod. Fertil. Dev..

[2] Rani P., Dutt R., Singh G., Chandolia R.K. (2018). Embryonic mortality in cattle—A review. Int. J. Curr. Microbiol. Appl. Sci..

[3] Hudson C. (2011). Understanding the factors affecting dairy cow fertility. Vet. Rec..

[4] Hudson C.D., Breen J.E., Bradley A.J., Green M.J. (2010). Fertility in UK dairy herds: Preliminary findings of a large-scale study. Cattle Pract..

[5] Walsh S.W., Williams E.J., Evans A.C. (2011). A review of the causes of poor fertility in high milk producing dairy cows. Anim. Reprod. Sci..

[6] Wathes D.C. (2012). Mechanisms linking metabolic status and disease with reproductive outcome in the dairy cow. Reprod. Domest. Anim..

[7] De Vries A. (2006). Economic value of pregnancy in dairy cattle. J. Dairy Sci..

[8] Forde N., Carter F., Spencer T.E., Bazer F.W., Sandra O., Mansouri-Attia N., Okumu L.A., McGettigan P.A., Mehta J.P., McBride R. (2011). Conceptus-induced changes in the endometrial transcriptome: How soon does the cow know she is pregnant?. Biol. Reprod..

[9] Diskin M.G., Waters S.M., Parr M.H., Kenny D.A. (2016). Pregnancy losses in cattle: Potential for improvement. Reprod. Fertil. Dev..

[10] Lonergan P., Forde N. (2014). Maternal-embryo interaction leading up to the initiation of implantation of pregnancy in cattle. Animal.

[11] Bazer F.W. (2013). Pregnancy recognition signaling mechanisms in ruminants and pigs. J. Anim. Sci. Biotechnol..

[12] Dorniak P., Bazer F.W., Wu G., Spencer T.E. (2012). Conceptus-derived prostaglandins regulate endometrial function in sheep. Biol. Reprod..

[13] Spencer T.E., Forde N., Dorniak P., Hansen T.R., Romero J.J., Lonergan P. (2013). Conceptus-derived prostaglandins regulate gene expression in the endometrium prior to pregnancy recognition in ruminants. Reproduction.

[14] Hansen P.J. (2011). The immunology of early pregnancy in farm animals. Reprod. Domest. Anim..

[15] Fray M.D., Paton D.J., Alenius S. (2000). The effects of bovine viral diarrhoea virus on cattle reproduction in relation to disease control. Anim. Reprod. Sci..

[16] Ridpath J.F. (2010). Bovine viral diarrhea virus: Global status. Vet. Clin. N. Am. Food Anim. Pract..

[17] Richter V., Kattwinkel E., Firth C.L., Marschik T., Dangelmaier M., Trauffler M., Obritzhauser W., Baumgartner W., Kasbohrer A., Pinior B. (2019). Mapping the global prevalence of bovine viral diarrhoea virus infection and its associated mitigation programmes. Vet. Rec..

[18] Scharnbock B., Roch F.F., Richter V., Funke C., Firth C.L., Obritzhauser W., Baumgartner W., Kasbohrer A., Pinior B. (2018). A meta-analysis of bovine viral diarrhoea virus (BVDV) prevalences in the global cattle population. Sci. Rep..

[19] Richter V., Lebl K., Baumgartner W., Obritzhauser W., Kasbohrer A., Pinior B. (2017). A systematic worldwide review of the direct monetary losses in cattle due to bovine viral diarrhoea virus infection. Vet. J..

[20] Grooms D.L. (2004). Reproductive consequences of infection with bovine viral diarrhea virus. Vet. Clin. N. Am. Food Anim. Pract..

[21] Wathes D.C., Oguejiofor C.F., Thomas C., Cheng Z. (2019). Importance of viral disease in dairy cow fertility. Engineering.

[22] Lanyon S.R., Hill F.I., Reichel M.P., Brownlie J. (2014). Bovine viral diarrhoea: Pathogenesis and diagnosis. Vet. J..

[23] Cheng Z., Abudureyimu A., Oguejiofor C.F., Ellis R., Barry A.T., Chen X., Anstaett O.L., Brownlie J., Wathes D.C. (2016). BVDV alters uterine prostaglandin production during pregnancy recognition in cows. Reproduction.

[24] Cheng Z., Chauhan L., Barry A.T., Abudureyimu A., Oguejiofor C.F., Chen X., Wathes D.C. (2017). Acute bovine viral diarrhea virus infection inhibits expression of interferon tau-stimulated genes in bovine endometrium. Biol. Reprod..

[25] Darnell J.E., Kerr I.M., Stark G.R. (1994). Jak-STAT pathways and transcriptional activation in response to IFNs and other extracellular signaling proteins. Science.

[26] Pestka S. (1997). The interferon receptors. Semin. Oncol..

[27] Bazer F.W., Ying W., Wang X., Dunlap K.A., Zhou B., Johnson G.A., Wu G. (2015). The many faces of interferon tau. Amino Acids.

[28] Vilcek S., Herring A.J., Herring J.A., Nettleton P.F., Lowings J.P., Paton D.J. (1994). Pestiviruses isolated from pigs, cattle and sheep can be allocated into at least three genogroups using polymerase chain reaction and restriction endonuclease analysis. Arch. Virol..

[29] Oguejiofor C.F., Cheng Z., Abudureyimu A., Fouladi-Nashta A.A., Wathes D.C. (2015). Global transcriptomic profiling of bovine endometrial immune response in vitro. I. Effect of lipopolysaccharide on innate immunity. Biol. Reprod..

[30] Oguejiofor C.F., Cheng Z., Abudureyimu A., Anstaett O.L., Brownlie J., Fouladi-Nashta A.A., Wathes D.C. (2015). Global transcriptomic profiling of bovine endometrial immune response in vitro. II. Effect of bovine viral diarrhea virus on the endometrial response to lipopolysaccharide. Biol. Reprod..

[31] Hansen T.R., Sinedino L.D.P., Spencer T.E. (2017). Paracrine and endocrine actions of interferon tau (IFNT). Reproduction.

[32] Aaronson D.S., Horvath C.M. (2002). A road map for those who don’t know JAK-STAT. Science.

[33] Chill J.H., Quadt S.R., Levy R., Schreiber G., Anglister J. (2003). The human type I interferon receptor: NMR structure reveals the molecular basis of ligand binding. Structure.

[34] Antonczyk A., Krist B., Sajek M., Michalska A., Piaszyk-Borychowska A., Plens-Galaska M., Wesoly J., Bluyssen H.A.R. (2019). Direct inhibition of IRF-dependent transcriptional regulatory mechanisms associated with disease. Front. Immunol..

[35] Gilbert R.O. (2011). The effects of endometritis on the establishment of pregnancy in cattle. Reprod. Fertil. Dev..

[36] Zhang Y., Mao D., Roswit W.T., Jin X., Patel A.C., Patel D.A., Agapov E., Wang Z., Tidwell R.M., Atkinson J.J. (2015). PARP9-DTX3L ubiquitin ligase targets host histone H2BJ and viral 3C protease to enhance interferon signaling and control viral infection. Nat. Immunol..

[37] Shuai K., Liu B. (2005). Regulation of gene-activation pathways by PIAS proteins in the immune system. Nat. Rev. Immunol..

[38] Sharrocks A.D. (2006). PIAS proteins and transcriptional regulation-more than just SUMO E3 ligases?. Genes Dev..

[39] Kouchaki E., Nikoueinejad H., Akbari H., Azimi S., Behnam M. (2019). The investigation of relevancy between PIAS1 and PIAS2 gene expression and disease severity of multiple sclerosis. J. Immunoass. Immunochem..

[40] Sadler A.J., Williams B.R. (2008). Interferon-inducible antiviral effectors. Nat. Rev. Immunol..

[41] Sheldon I.M., Cronin J., Goetze L., Donofrio G., Schuberth H.J. (2009). Defining postpartum uterine disease and the mechanisms of infection and immunity in the female reproductive tract in cattle. Biol. Reprod..

[42] Peterhans E., Schweizer M. (2013). BVDV: A pestivirus inducing tolerance of the innate immune response. Biologicals.

[43] Iqbal M., Poole E., Goodbourn S., McCauley J.W. (2004). Role for bovine viral diarrhea virus Erns glycoprotein in the control of activation of beta interferon by double-stranded RNA. J. Virol..

[44] Chen Z., Rijnbrand R., Jangra R.K., Devaraj S.G., Qu L., Ma Y., Lemon S.M., Li K. (2007). Ubiquitination and proteasomal degradation of interferon regulatory factor-3 induced by Npro from a cytopathic bovine viral diarrhea virus. Virology.

[45] Hilton L., Moganeradj K., Zhang G., Chen Y.H., Randall R.E., McCauley J.W., Goodbourn S. (2006). The NPro product of bovine viral diarrhea virus inhibits DNA binding by interferon regulatory factor 3 and targets it for proteasomal degradation. J. Virol..

[46] Schweizer M., Matzener P., Pfaffen G., Stalder H., Peterhans E. (2006). “Self” and “nonself” manipulation of interferon defense during persistent infection: Bovine viral diarrhea virus resists alpha/beta interferon without blocking antiviral activity against unrelated viruses replicating in its host cells. J. Virol..

[47] Shoemaker M.L., Smirnova N.P., Bielefeldt-Ohmann H., Austin K.J., van Olphen A., Clapper J.A., Hansen T.R. (2009). Differential expression of the type I interferon pathway during persistent and transient bovine viral diarrhea virus infection. J. Interferon Cytokine Res..

[48] Smirnova N.P., Bielefeldt-Ohmann H., Van Campen H., Austin K.J., Han H., Montgomery D.L., Shoemaker M.L., van Olphen A.L., Hansen T.R. (2008). Acute non-cytopathic bovine viral diarrhea virus infection induces pronounced type I interferon response in pregnant cows and fetuses. Virus Res..

[49] Hansen T.R., Smirnova N.P., Webb B.T., Bielefeldt-Ohmann H., Sacco R.E., Van Campen H. (2015). Innate and adaptive immune responses to in utero infection with bovine viral diarrhea virus. Anim. Health Res. Rev..

[50] Xie P. (2013). TRAF molecules in cell signaling and in human diseases. J. Mol. Signal..

[51] Wathes D.C., Lamming G.E. (1995). The oxytocin receptor, luteolysis and the maintenance of pregnancy. J. Reprod. Fertil. Suppl..

[52] Dorniak P., Bazer F.W., Spencer T.E. (2013). Physiology and Endocrinology Symposium: Biological role of interferon tau in endometrial function and conceptus elongation. J. Anim. Sci..

