# Supplementary material for: Bovine Viral Diarrhoea Virus Infection Disrupts Uterine Interferon Stimulated Gene Regulatory Pathways During Pregnancy Recognition in Cows
Source: Viruses. 2019 Dec 18;12(1):1. doi: 10.3390/v12010001 (PMC7020065; doi:10.3390/v12010001)

Supplementary file 2

Diagnostic plots for ANOVA with linear mixed effect model

1. IFNAR1

1.1. Observed by predicted plot

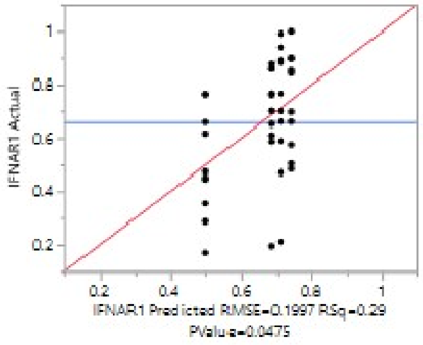

1.2. Residual by predicted plot

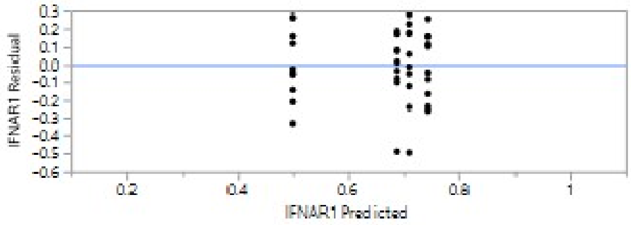

1.3. Treatment plot

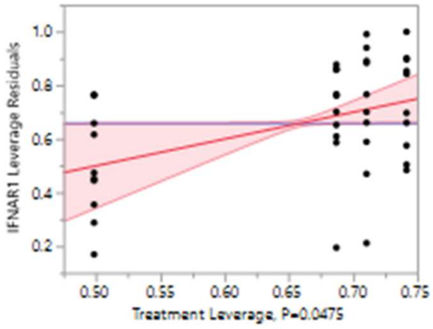

1.4. Histogram

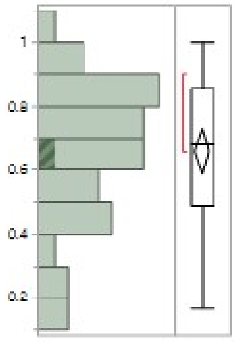

2. IFNAR2

2.1. Observed by predicted plot

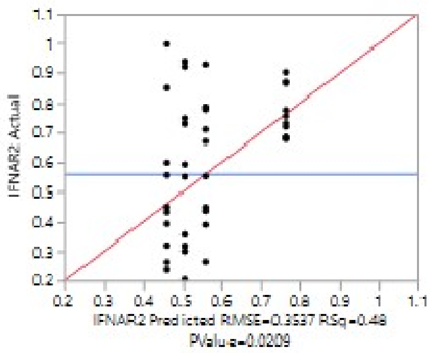

2.2. Residual by predicted plot

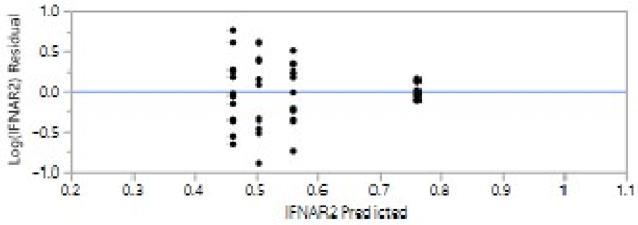

2.3. Treatment leverage plot

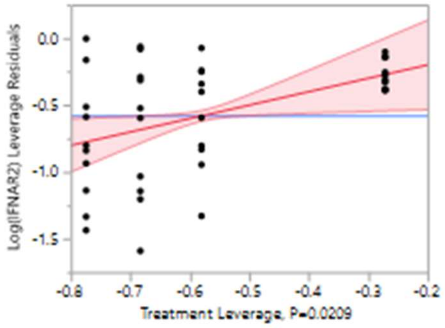

2.4. Histogram

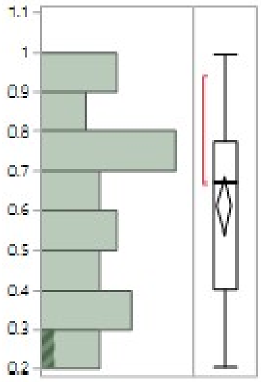

3. JAK1

3.1. Observed by predicted plot

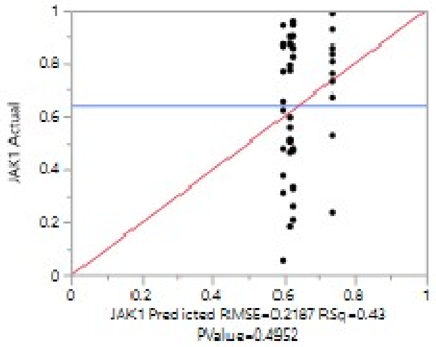

3.2. Residual by predicted plot

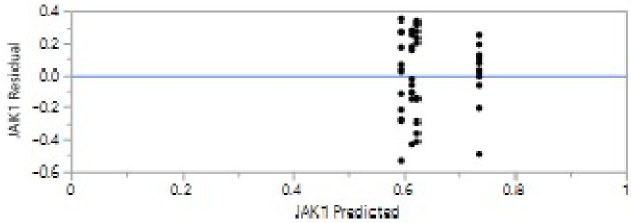

3.3. Treatment leverage plot

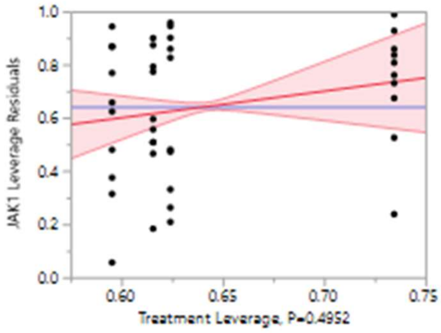

3.4. Histogram

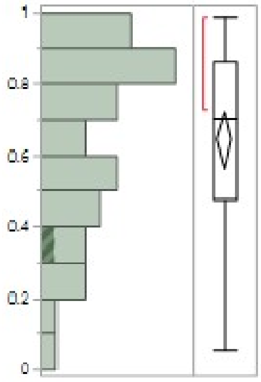

4. TYK2

4.1. Observed by predicted plot

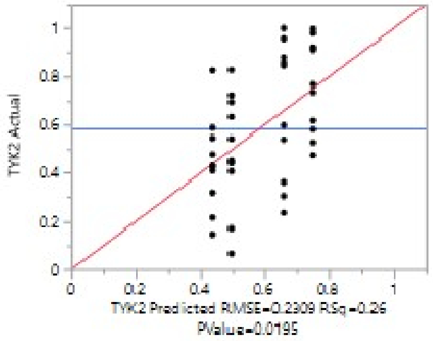

4.2. Residual by predicted plot

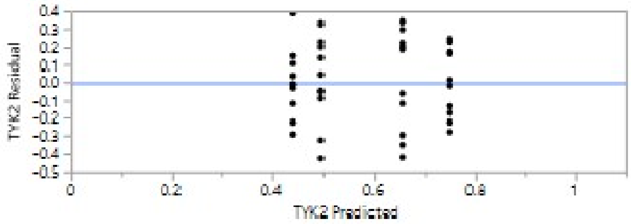

4.3. Treatment leverage plot

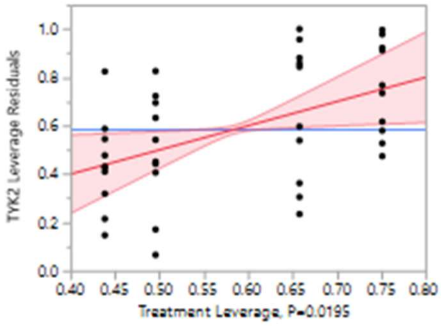

4.4. Histogram

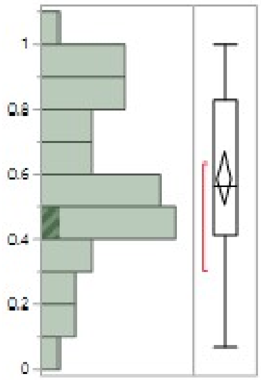

5. IRF7

5.1. Observed by predicted plot

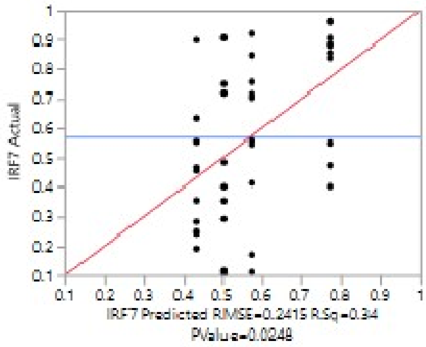

5.2. Residual by predicted plot

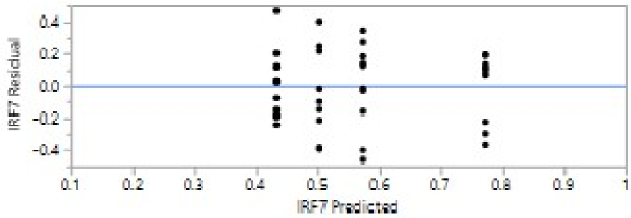

5.3. Treatment leverage plot

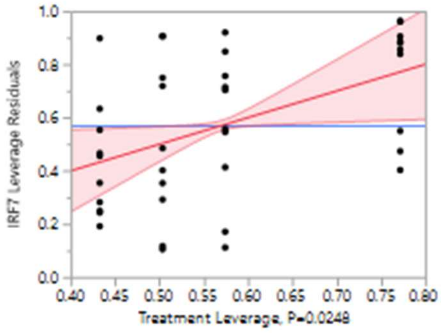

5.4. Histogram

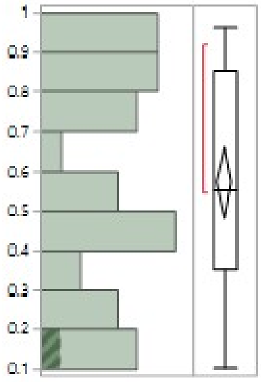

6. IRF9

6.1. Observed by predicted plot

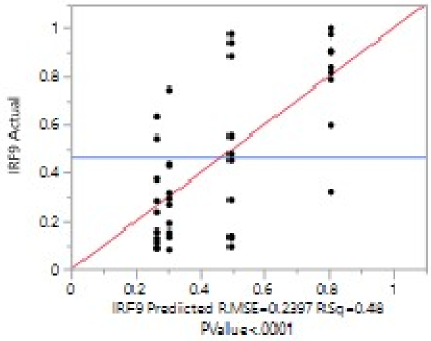

6.2. Residual by predicted plot

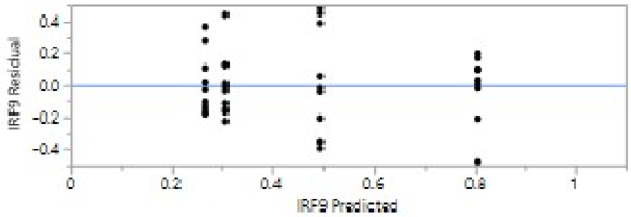

6.3. Treatment leverage plot

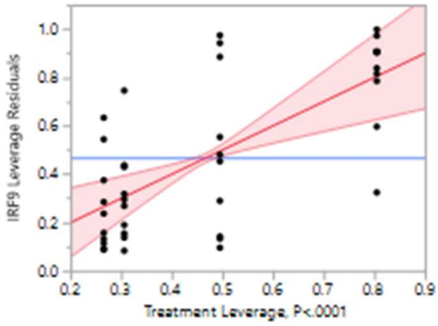

6.4. Histogram

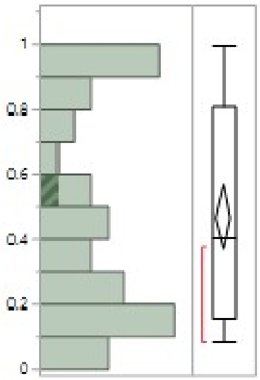

7. STAT1

7.1. Observed by predicted plot

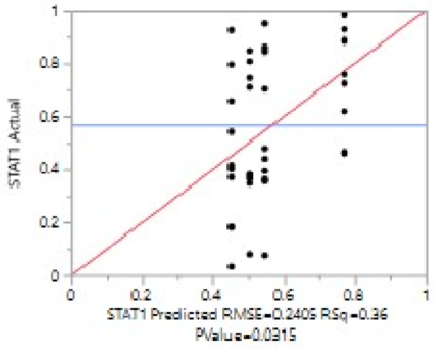

7.2. Residual by predicted plot

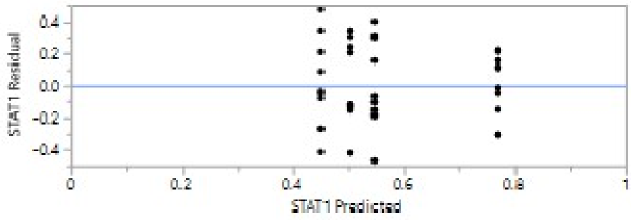

7.3. Treatment leverage plot

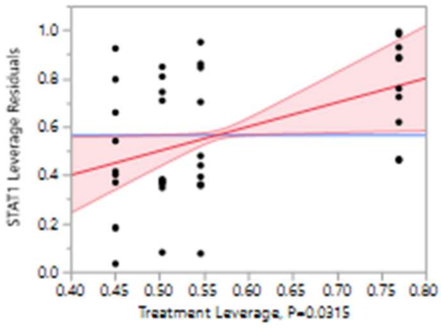

7.4. Histogram

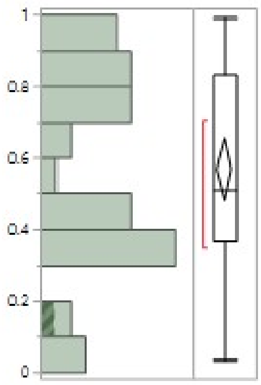

8. STAT2

8.1. Observed by predicted plot

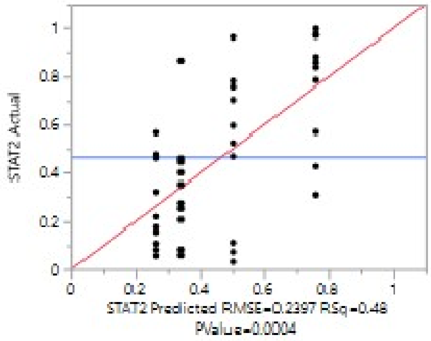

8.2. Residual by predicted plot

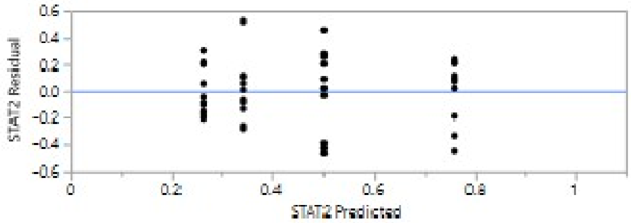

8.3. Treatment leverage plot

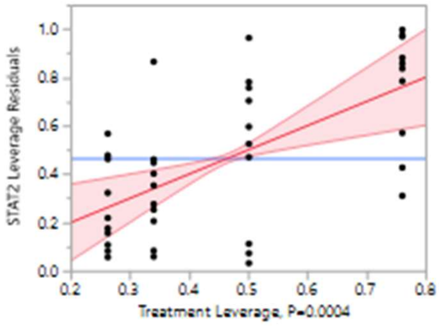

8.4. Histogram

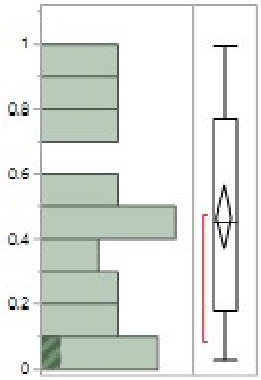

9. IFNG

9.1. Observed by predicted plot

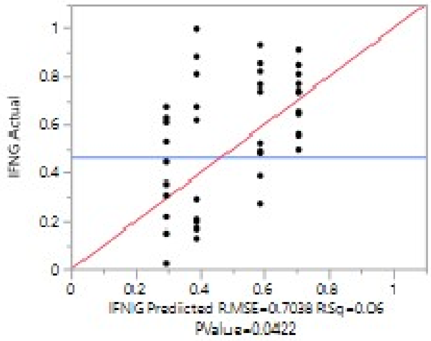

9.2. Residual by predicted plot

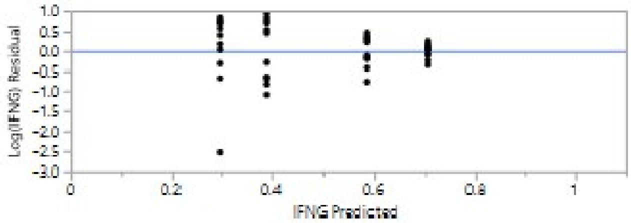

9.3. Treatment leverage plot

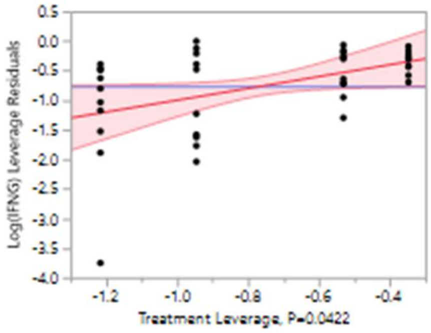

9.4. Histogram

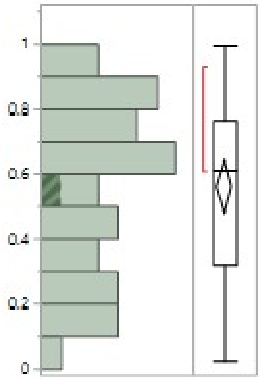

10. PIAS2

10.1. Observed by predicted plot

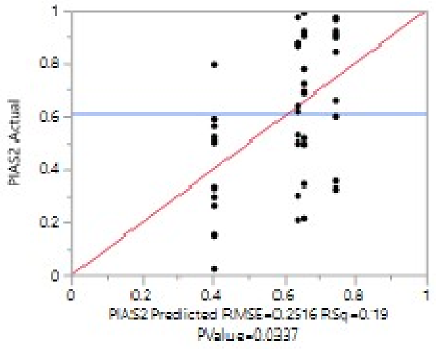

10.2. Residual by predicted plot

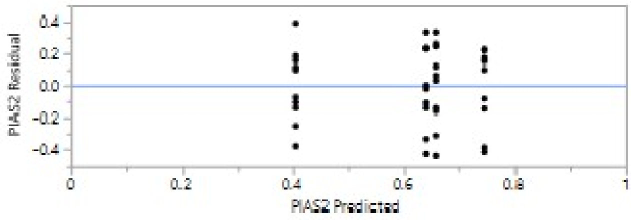

10.3. Treatment leverage plot

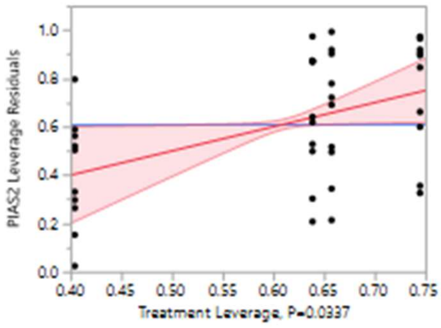

10.4. Histogram

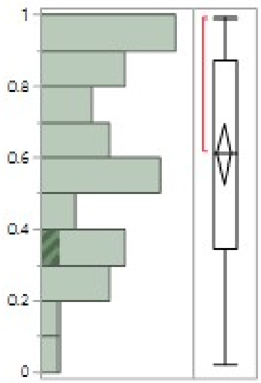

11. STAT2 Protein

11.1. Observed by predicted plot

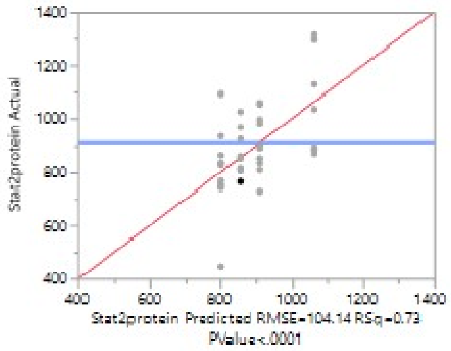

11.2. Residual by predicted plot

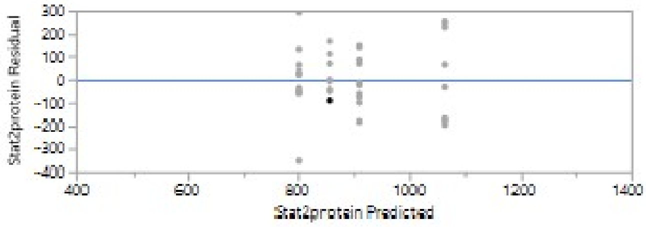

11.3. Treatment leverage plot

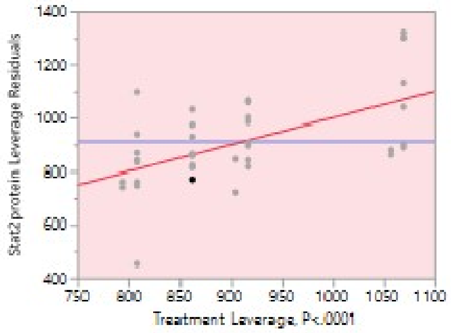

11.4. Histogram

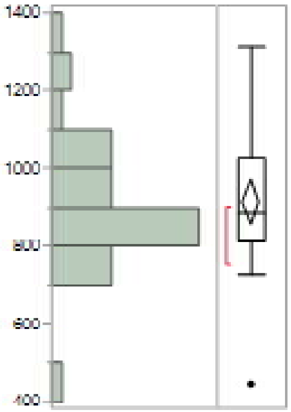

Supplement: Supplementary file 1 [file viruses-12-00001-s001.zip › viruses-658834.suppl zip/S2.pdf]
