# Supplementary material for: Bovine Viral Diarrhoea Virus Infection Disrupts Uterine Interferon Stimulated Gene Regulatory Pathways During Pregnancy Recognition in Cows
Source: Viruses. 2019 Dec 18;12(1):1. doi: 10.3390/v12010001 (PMC7020065; doi:10.3390/v12010001)
Supplement: Supplementary file 1 [file viruses-12-00001-s001.zip › viruses-658834.suppl zip/S3.pdf]

Supplementary file 3

SPSS output of Variance components via linear mixed effect model

Genes	Estimate	Std error	Wald Z	P values
IFNAR1				
Residual	0.0396	0.0108	3.6740	0.0000
Cow variance	0.0311	0.0067	0.4630	0.6430
IFNAR2				
Residual	0.0236	0.0064	3.674	0.0000
Cow variance	0.0076	0.0065	1.1610	0.2460
JAK1				
Residual	0.0478	0.0130	3.6740	0.0000
Cow variance	0.1942	0.0151	1.2820	0.0000
TYK2				
Residual	0.0533	0.01450	3.6740	0.0000
Cow variance	0.0006	0.0074	0.0790	0.9370
IRF7				
Residual	0.0583	0.0158	3.6740	0.0000
Cow variance	0.0059	0.0104	0.5690	0.5690
IRF9				
Residual	0.0574	0.0156	3.6740	0.0000
Cow variance	0.0009	0.0081	0.108	0.914
STAT1				
Residual	0.5784	0.1574	3.6740	0.0000
Cow variance	0.0079	0.0112	0.7060	0.4800
STAT2				
Residual	0.5744	0.0156	3.6740	0.0000
Cow variance	0.0056	0.0102	0.556	0.578
IFNG				
Residual	0.0863	0.0203	4.230	0.0000
Cow variance	N/A	N/A	N/A	N/A
PIAS2				
Residual	0.0732	0.0199	3.674	0.0000
Cow variance	0.0067	0.0128	0.5280	0.597
STAT2 Protein				
Residual	10844.27	3071.08	3.5310	0.0000
Cow variance	10318.69	6310.99	1.635	0.102
