# Supplementary material for: Bovine Viral Diarrhoea Virus Infection Disrupts Uterine Interferon Stimulated Gene Regulatory Pathways During Pregnancy Recognition in Cows
Source: Viruses. 2019 Dec 18;12(1):1. doi: 10.3390/v12010001 (PMC7020065; doi:10.3390/v12010001)
Supplement: Supplementary file 1 [file viruses-12-00001-s001.zip › viruses-658834.suppl zip/S4.pdf]

Supplementary file 4

Test of homogeneity of variance

Gene Name	Levene' statistic*	P values
IFNAR1	0.229	0.876
IFNAR2	3.596	0.023
STAT1	0.615	0.610
STAT2	1.256	0.304
IRF7	0.875	0.463
IRF9	2.161	0.110
JAK1	1.767	0.171
TYK2	1.677	0.189
PIAS2	0.059	0.981
IFNG	7.290	0.001
STAT2 Protein	1.809	0.244

* Levene's statistic based on mean
